# Cortisol, cytokines, and hippocampal volume interactions in the elderly

**DOI:** 10.3389/fnagi.2014.00153

**Published:** 2014-07-03

**Authors:** Keith D. Sudheimer, Ruth O'Hara, David Spiegel, Bevin Powers, Helena C. Kraemer, Eric Neri, Michael Weiner, Antonio Hardan, Joachim Hallmayer, Firdaus S. Dhabhar

**Affiliations:** ^1^Department of Psychiatry and Behavioral Sciences, Stanford University School of Medicine, Stanford UniversityStanford, CA, USA; ^2^Department of Radiology, University of CaliforniaSan Francisco, CA, USA; ^3^Department of Veterans Affairs Medical Center, Center for Imaging of Neurodegenerative DiseasesSan Francisco, CA, USA; ^4^Institute for Immunity, Transplantation, and Infection, Stanford UniversityStanford, CA, USA

**Keywords:** cytokines, cortisol, hippocampus, cognition, aging

## Abstract

Separate bodies of literature report that elevated pro-inflammatory cytokines and cortisol negatively affect hippocampal structure and cognitive functioning, particularly in older adults. Although interactions between cytokines and cortisol occur through a variety of known mechanisms, few studies consider how their interactions affect brain structure. In this preliminary study, we assess the impact of interactions between circulating levels of IL-1Beta, IL-6, IL-8, IL-10, IL-12, TNF-alpha, and waking cortisol on hippocampal volume. Twenty-eight community-dwelling older adults underwent blood draws for quantification of circulating cytokines and saliva collections to quantify the cortisol awakening response. Hippocampal volume measurements were made using structural magnetic resonance imaging. Elevated levels of waking cortisol in conjunction with higher concentrations of IL-6 and TNF-alpha were associated with smaller hippocampal volumes. In addition, independent of cortisol, higher levels of IL-1beta and TNF-alpha were also associated with smaller hippocampal volumes. These data provide preliminary evidence that higher cortisol, in conjunction with higher IL-6 and TNF-alpha, are associated with smaller hippocampal volume in older adults. We suggest that the dynamic balance between the hypothalamic-pituitary adrenal axis and inflammation processes may explain hippocampal volume reductions in older adults better than either set of measures do in isolation.

## Introduction

Cytokines are small proteins, which are produced by a variety of immune cells (lymphocytes, macrophages, natural killer cells etc.) and are critically important for cell signaling during when the immune system is mounting inflammatory responses to infection and cell damage. Cortisol is an adrenal glucocorticoid hormone produced in the zona fasciculate of the adrenal cortex and has a critical role in several physiological systems including acting as a anti-inflammatory agent.

Glucocorticoids and several cytokines have been associated with decreased hippocampal volumes and associated cognitive impairments. Endogenous cortisol levels have consistently been shown to predict smaller hippocampal volumes, faster hippocampal atrophy, and memory deficits (Lupien et al., 1998; O'Hara, 2007; O'Hara et al., 2007). Exogenous glucocorticoids can also adversely affect both hippocampal morphology and function during cognitive tasks (Hsu et al., 2002; de Quervain et al., 2003; Brown et al., 2004; Abercrombie et al., 2011) in humans, potentially by inhibiting neurogenesis and/or increasing the risk of cell death (Woolley et al., 1990; Sapolsky, 2000; Hsu et al., 2002). Specific cytokines e.g., IL-6, have been found to be inversely correlated with hippocampal gray matter volumes (Tanabe et al., 1997; Marsland et al., 2008) and can adversely affect hippocampal morphology during development in animal models (Kraemer and Blasey, 2004; Samuelsson et al., 2006). Both IL-1Beta and TNF-Alpha have also been shown to inhibit long-term potentiation and synaptic plasticity in the hippocampus and potentially contribute to excitotoxicity (Marques-Deak et al., 2005; Hermann et al., 2006; Pickering and O'Connor, 2007). In humans TNF-alpha single nucleotide polymorphisms predict smaller hippocampal volumes (Chapman et al., 1987; Spath-Schwalbe et al., 1998; Baune et al., 2012).

Glucocorticoids and cytokines exert their effects on the brain by acting directly on receptors and indirectly through a variety of mechanisms such as neurotransmission, intracellular signaling, and subsequent gene expression. Cortisol receptors are expressed in the hippocampus and many other cortical and subcortical regions of the humans brain (Sarrieau et al., 1986; Jonat et al., 1990; Pariante et al., 1999; Pace et al., 2007). Cortisol can penetrate the blood brain barrier in order to bind to these receptors (Woolley et al., 1990; Sapolsky, 2000; Pariante et al., 2004). Similarly, for cytokines there is evidence from both human and animal studies that IL-1, IL-6, and TNF-alpha protein and/or receptors are expressed in hippocampus (Schöbitz et al., 1994; Banks et al., 1995; Chandler et al., 1997; Vitkovic et al., 2000a,b; Quan et al., 2003; Reiche et al., 2004; Yamamoto et al., 2006; Yirmiya and Goshen, 2011). Furthermore, microglia located throughout the brain express IL-1Beta, IL-6, IL-8, IL-10, IL-12, IL-15, and TNF-alpha as well as the IL-1R1 (receptor) (Lee et al., 2002) which interacts with IL-1Beta and is dynamically expressed in rat hippocampal neurons.

The biological actions of pro-inflammatory cytokines and glucocorticoids are not independent of each other and interact on multiple levels. Chronic exposure to glucocorticoids are known to inhibit the immune system cells responsible for producing peripheral cytokines (Dhabhar, 2009). Glucocorticoids can exert broad anti-inflammatory effects by inhibiting the transcription and action of many of the pro-inflammatory cytokines including IL-1Beta, IL-6, TNF-Alpha, and others (see Webster et al., 2002 for a review). Cytokines can also exert influence over glucocorticoid secretion, bioavailability, and signaling. IL-1Beta and IL-6 can activate the HPA axis (Berkenbosch et al., 1987; Mastorakos et al., 1993; Shintani et al., 1995; Besedovsky and del Rey, 2000) directly. Pro-inflammatory cytokines that produce fever can indirectly dislodge significant amounts of biologically inactivated glucocorticoids from corticosteroid binding globulin in the blood (Cameron et al., 2010) making cortisol more available to target tissues. In addition IL-1 and TNF-Alpha can impair cortisol signaling by interfering with glucocorticoid receptor phosphorylation and interactions between the ligand-receptor complex and glucocorticoid response elements (see Pace et al., 2007 for review).

Despite many known parallels between cytokine and glucocorticoid influence on the hippocampus, and known physiological interactions between these systems, relatively few studies have systematically addressed the issue of interactions between cytokines and glucocorticoids on the level of gross hippocampal morphology. To address this gap in the literature the current study was designed to characterize the independent and interactive effects of endogenous levels of cortisol and several major cytokines (IL-1Beta, IL-6, IL-8, IL-10, IL-12, TNF-alpha), on hippocampal volume in the elderly.

## Methods and materials

Participants included 28 community-dwelling older adults (15 females and 13 males) between 61 and 100 years of age (mean = 69.0, *SD* = 8.6). Participants were recruited through advertisements, contacts with local senior centers, or had previously participated in studies at Stanford University. All participants provided written informed consent for their participation in accordance with the Stanford University internal review board's regulations. All participants filled out questionnaires relating to their current medical conditions and medical history. They also underwent the Mini-Mental State Exam (MMSE) (Folstein et al., 1975) and the Structured Clinical Interview for DSM-IV-TR (SCID). Individuals with a MMSE less than 26 or with any Axis I psychiatric disorders were excluded. Participants were also excluded if they were currently using any systemic corticosteroids, psychotropic medications, short-acting anxiolytics, sedative hypnotics, or medications with significant impact on cytokine levels, cholinergic, or anticholinergic side effects, as well as any FDA-approved medications for the treatment of Alzheimer's disease. Some participants had a prior history of high blood pressure, or other cardiovascular issues, all of which were stabilized or resolved at the time of the study. Those individuals with unresolved health issues were not included in the study.

Upon entry into the study, all participants had whole blood drawn at the Stanford University Clinical Translational Research Unit for assessment of circulating cytokine levels. Whole-blood was collected into 10 ml SST tubes (Becton Dickinson, Franklin Lakes, NJ). Serum was allowed to clot for 30 min at room temperature, then spun (1300 rpm for 15 min), frozen, and stored at −75°C for subsequent cytokine quantification. A high sensitivity multiplexed sandwich immunoassay was used to quantify IL-1Beta, IL-6, IL-8, IL-10, IL-12, and TNF-alpha, concentrations (Mesoscale Discovery, Gaithersburg, MD). The intra-assay coefficients of variation were: IL-1Beta (2.7%), IL-6 (4.4%), IL-8 (9.8%), IL-10 (3.1%), IL-12 (7.0%), and. TNF-alpha (4.2%), The inter-assay coefficients of variation were: IL-1Beta (3.0%), IL-6 (5.5%), IL-8 (6.2%), IL-10 (4.9%), IL-12 (8.7%), and. TNF-alpha (3.0%), The sensitivity of each cytokine assay conducted was IL-1Beta (1.5 pg/ml), IL-6 (0.4 pg/ml), IL-8 (0.4 pg/ml), IL-10 (0.6 pg/ml), IL-12 (2.4 pg/ml), and TNF-alpha (0.4 pg/ml).

Participants also completed a salivary cortisol measurement protocol over 2 consecutive days in their own home. Each participant was scheduled to obtain saliva samples at the time of waking, 30 min later, and then at 1200, 1700, and 2100 h. Each participant was instructed to collect saliva using the Salivette system (Sarstedt, Inc., Newton, NC). Participants were instructed to refrigerate each sample immediately after collection. Participants then shipped samples to the Stanford Clinical and Translational Research Unit within a week of collection. Participants were instructed not to eat, drink, smoke, brush their teeth, or use mouthwash within the 30 min before collection and not to drink alcohol during the 8–10 h prior to collecting samples or during the 2 days of collection. No physical saliva stimulants were used. Our prior work compared actual times recorded by participants at each saliva collection with a Medication Event Monitoring Units (MEMS) log and indicated that older adults were highly compliant with the protocol. Samples were stored at −80°C prior to analysis. Samples were then centrifuged and analyzed for salivary cortisol using luminescence immunoassay (LIA) reagents provided by Immuno-Biological Laboratories, Inc. (Hamburg, Germany). Assay sensitivity was 0.015 μg/dl. Intra-assay variation on three saliva pools of the low, medium, and high controls were averaged 2.78, 10.45, and 4.79%, respectively. The mean values of the low, medium, and high controls were 0.054, 0.228, and 0.863 μg/dl. The inter-assay coefficients of variation for the low, medium, and high controls were 10.9, 10.5, and 5.5%, respectively.

All MRI scans were performed at the Palo Alto VAHCS on a General Electric Signa 1.5 Tesla clinical MRI scanner with a standard radio frequency head coil. Two high resolution anatomical scans were collected: a Proton density and T2-weighted spin-echo MRI, *TR*/*TE*1/*TE*2 = 5000/30/80 ms, 51 contiguous axial slices (3 mm thickness) covering the entire brain and angulated parallel to the long axis of the hippocampal formation, 0.976 × 0.976 mm^2^ in-plane resolution (acquisition time = 17 min); and a Three-dimensional spoiled GRASS (SPGR) MRI of entire brain, *TR*/*TE* = 9/2 ms, 15° flip angle, perpendicular to the long axis of the hippocampus, 0.976 × 0.976 mm^2^ in-plane resolution, 1.5 mm slice thickness, no skip (acquisition time = 7 min 27 s).

Hippocampal volume measurements were carried out using a semi-automatic, commercially available, high dimensional brain-mapping tool (Medtronic Surgical Navigation Technologies, Louisville, CO). Our group (O'Hara, 2007) and others (Hsu et al., 2002) have independently validated this method, with high levels of inter-rater reliability, comparing it with manual hippocampal tracing in patient populations and healthy participants. Manual and semi-automated volume measurements of the hippocampus generally correlated better than *r* = 0.9 (Hsu et al., 2002). However, the semi-automated method depends less on rater judgment and manual tracing, reducing potential rater bias. All hippocampal boundaries were visually reviewed on each brain MRI. If faulty registrations of the hippocampal boundaries were found, manual corrections were made. No MRI scans had to be discarded as a result. To adjust for differences in head size, all hippocampal volumes were normalized to total intracranial volume (TIV). TIV was identified using a mask containing the brain as identified on the T2-weighted image, as described previously (Tanabe et al., 1997). Due to inter-subject differences in brain coverage during acquisition, we standardized TIV by excluding all slices below the level of the inferior aspect of the temporal lobe from the intracranial volume measurement.

In order to test the interactions amongst cytokines, waking cortisol levels, and hippocampal volumes, we employed a multiple regression approach. For each cytokine we constructed an independent multiple regression model that included waking cortisol, age, and sex as covariates and hippocampal volume as the outcome variable. All measures were entered as continuous measures with the exception of sex. The main effects of each of the cytokines and their interaction with hippocampal volume were tested in these models. All independent variables were centered in accordance with best statistical practices (Kraemer and Blasey, 2004). Cortisol and cytokine values were also log-transformed, as these measures often have skewed distributions. After log transformation simple correlations were also used to further describe the pairwise relationships amongst cytokines, cortisol, and hippocampal volume.

We stress that this is an exploratory study aimed at identifying any potential relationships between cortisol, cytokines, and hippocampal volumes in an elderly population, in order to generate hypotheses that can be tested in independent follow up studies. The exploratory nature of our study is due to the lack of extant data to suggest which specific cytokines would be most likely to interact with cortisol on hippocampal volume.

## Results

We observed main effects of both cortisol and cytokines on hippocampal volume. High levels of IL-1Beta (β = −0.59, *p* = 0.026) and TNF-alpha (β = −0.44, *p* = 0.025) were associated with smaller hippocampal volumes. High levels of cortisol were associated with smaller hippocampal volumes when the effects of IL-8, IL-10, or TNF-alpha, were controlled for. However, when controlling for the other cytokines under investigation this the relationship between cortisol and hippocampal volume not achieve significance (See Table 1). A non-parametric Spearman's rank correlation also indicated only a trend level inverse relationship between cortisol and hippocampal volume (*r* = −0.33, *p* = 0.083).

**Table 1:** **The effects and interactions of various cytokines and cortisol levels on hippocampal volume (corrected for total intracranial volume)**.

**Outcome Measure**	**Test variable(s)**	**Statistic**	***P*-value**
Hippocampal volume	Omnibus	*r*^2^ = 0.49, *F*_(5, 22)_ = 4.21, *N* = 28	*p* = 0.008^*^
	TNFa^m^	β = −0.44	*p* = 0.025^*^
	Cortisol^m^	β = −0.37	*p* = 0.033^*^
	TNFa × Cortisol^i^	β = −0.43	*p* = 0.047^*^
Hippocampal volume	Omnibus	*r*^2^ = 0.62, *F*_(5, 17)_ = 5.46, *N* = 23	*p* = 0.004^*^
	IL-B^m^	β = −0.59	*p* = 0.026^*^
	Cortisol^m^	β = −0.25	*p* = 0.307
	IL-B × Cortisol^i^	β = −0.41	*p* = 0.173
Hippocampal volume	Omnibus	*r*^2^ = 0.46, *F*_(5, 22)_ = 3.73, *N* = 28	*p* = 0.014^*^
	IL-6^m^	β = −0.01	*p* = 0.943
	Cortisol^m^	β = −0.22	*p* = 0.217
	IL-6 × Cortisol^i^	β = −0.42	*p* = 0.037^*^
Hippocampal volume	Omnibus	*r*^2^ = 0.40, *F*_(5, 22)_ =2.92, *N* = 28	*p* = 0.036^*^
	IL-8^m^	β = −0.02	*p* = 0.929
	Cortisol^m^	β = −0.39	*p* = 0.039^*^
	IL-8 × Cortisol^i^	β = 0.29	*p* = 0.152
Hippocampal volume	Omnibus	*r*^2^ = 0.43, *F*_(5, 22)_ = 3.26, *N* = 28	*p* = 0.024^*^
	IL-10^m^	β = −0.33	*p* = 0.096
	Cortisol^m^	β = −0.43	*p* = 0.025^*^
	IL-10 × Cortisol^i^	β = −0.29	*p* = 0.169
Hippocampal volume	Omnibus	*r*^2^ = 0.38, *F*_(5, 22)_ = 2.68, *N* = 28	*p* = 0.049^*^
	IL-12^m^	β = −0.24	*p* = 0.198
	Cortisol^m^	β = −0.39	*p* = 0.051
	IL-12 × Cortisol^i^	β = −0.10	*p* = 0.628

Main effects^m^, interactions^i^ within multiple regression models are indicated for the hippocampal volume. All multiple regression models include covariates for age, sex, and cortisol. Beta estimates (β) are standardized. All multiple regression models test for the main effect the cytokine, the main effect of cortisol, and the interaction between cortisol and the cytokine on hippocampal volume.

* *= p < 0.05*.

**Figure 1:**
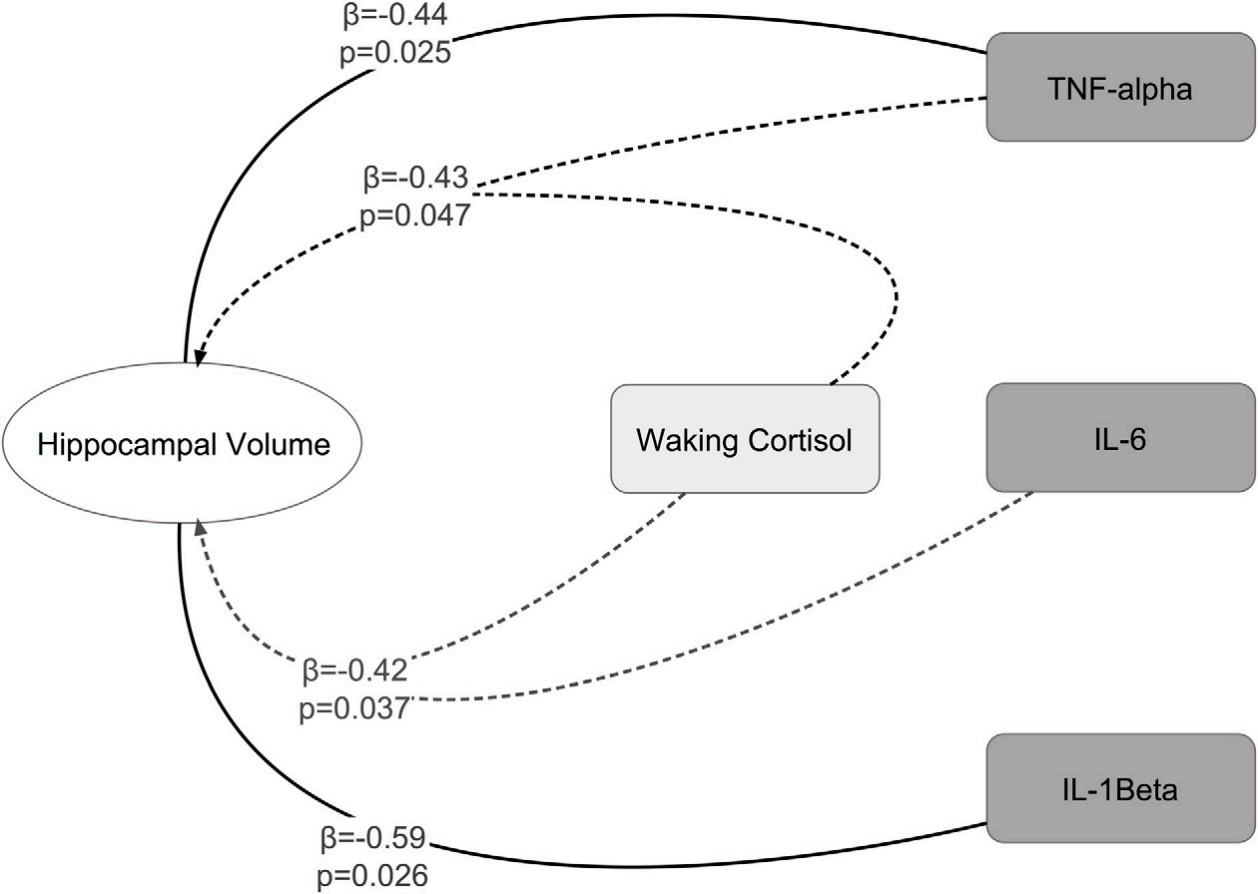
**Significant main effects and interactions between cytokines, waking cortisol, and hippocampal volume**.

**Figure 2:**
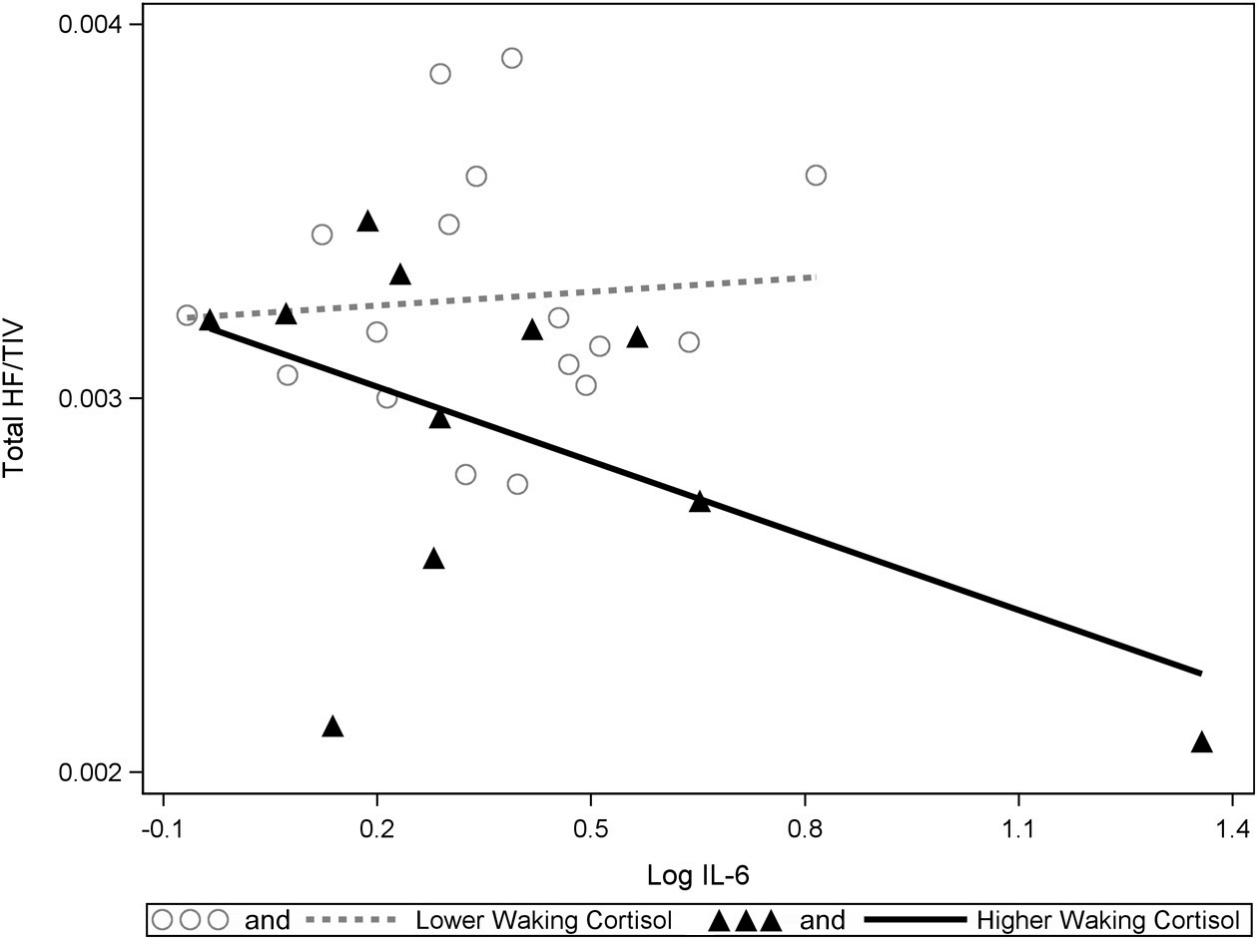
**A significant interaction between IL-6 and cortisol on hippocampal volumes**. Higher waking cortisol and concurrently high levels of IL-6 are associated with smaller hippocampal volumes. “Lower” and “higher” waking cortisol levels are displayed as a median split to allow the visualization of the multiple regression results. However, continuous variables were used in all multiple regressions models.

**Figure 3:**
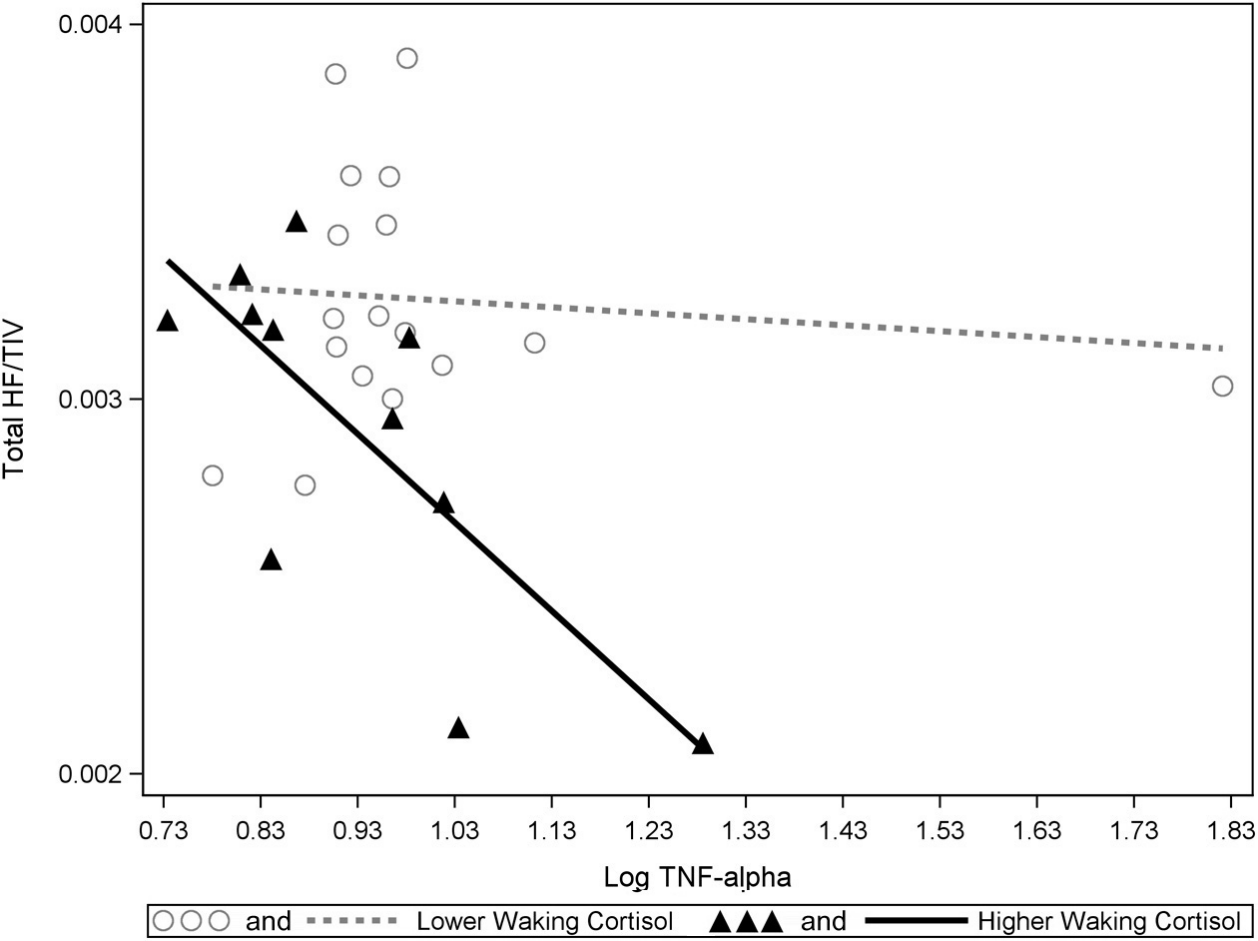
**A significant interaction between TNF-alpha and cortisol on hippocampal volumes**. Higher waking cortisol and concurrently high levels of TNF-alpha are associated with smaller hippocampal volumes. “Lower” and “higher” waking cortisol levels are displayed as a median split to allow the visualization of the multiple regression results. However, continuous variables were used in all multiple regressions models.

Significant interactions between cortisol and cytokine measures were also observed. These interactions were between cortisol and both IL-6 (β = −0.42, *p* = 0.037; See Figures 1, 2) and TNF-alpha (β = −0.43, *p* = 0.047; See Figures 1, 3). Having concurrently high cytokine levels and high waking cortisol levels was associated with smaller hippocampal volumes.

## Discussion

Our results suggest elevated levels of either IL-6 or TNF-alpha in combination with elevated waking cortisol is associated with reduced hippocampal volume in healthy older participants. This data also suggests that independent of the effects of cortisol, higher levels of either IL-1Beta or TNF-alpha are also associated with lower hippocampal volumes. One outlier was identified in the TNF-alpha data (far right in Figure 3). However, significant statistical interactions were observed with or without inclusion or exclusion of this outlier.

Cortisol and cytokines are both known to affect the hippocampus and to interact with each other via central and peripheral mechanisms. Cortisol is generally an inhibitory regulator of pro-inflammatory cytokine production (IL-1Beta, TNF-alpha) and action (Marques-Deak et al., 2005; Hermann et al., 2006). Cortisol is also released in reaction to the presence of pro-inflammatory cytokines (Chapman et al., 1987; Spath-Schwalbe et al., 1998). Several cytokines (IL-1Beta, IL-2, IL-4, TNF-alpha, IFN-α) also exert indirect inhibitory influence over glucocorticoid receptor translocation and signaling (Jonat et al., 1990; Pariante et al., 1999; Pace et al., 2007). These multiple levels of physiological interactions between cytokines and cortisol suggest that there are multiple potential explanations for the statistical cortisol and cytokine interactions that we observe. Two potential mechanisms may explain our findings; (1) a dual-toxicity hypothesis and (2) a peripheral cytokine activation of cortisol hypothesis.

Under the dual-toxicity hypotheses cortisol and various cytokines could be both adversely impacting hippocampal tissues resulting in volume decreases. Under the peripheral cytokine activation of cortisol hypotheses the same results could also be explained by a peripheral interaction between cytokines and cortisol. In this scenario, immune triggers could be repeatedly activating cytokines, which then induce increases in unbound cortisol. This unbound cortisol can then cross the blood-brain-barrier and affects the hippocampus as well as other HPA axis regulatory mechanisms. Repeated immune challenges and subsequent cortisol release could also be an underlying mechanism for hippocampal atrophy.

Some evidence suggests that a central dual toxicity interaction is possible. Hypercortisolemia has been shown to negatively impact neuronal function in the hippocampus in a range of animal studies. Specifically there is evidence that glucocorticoids can alter dendritic morphology, inhibit neurogenesis, and increase the risk of cell death (Woolley et al., 1990; Sapolsky, 2000).

Despite a limited ability to cross the blood brain barrier previous studies suggest that peripheral cytokines can still exert influence on central targets. These effects may be accomplished via degradation of the blood-brain-barrier, active transport into the brain, activation of central nervous system cytokines via neural signals mediated through endothelial cells astrocytes and glial cells (Banks et al., 1995; Chandler et al., 1997; Quan et al., 2003; Reiche et al., 2004; Yirmiya and Goshen, 2011). While less is known regarding the impact of cytokines on hippocampus, pro-inflammatory cytokines acting centrally can have many deleterious effects. TNF-alpha in particular has been shown to contribute to neuronal (Barker et al., 2001; McGuire, 2001) and oligodendroglia cell death (Buntinx et al., 2004a,b; Li et al., 2008) and to decrease various neurotrophic support mechanisms that contribute to cell survival (Barker et al., 2001).

Alternatively there are also mechanisms by which peripheral cytokine actions could drive systemic hypercortisolemia, which effects on the hippocampus. Peripheral cytokine actions can directly activate the HPA axis, interfere with cortisol receptor signaling and indirectly dislodge cortisol from its binding proteins. These processes can dramatically increase the amount of cortisol disrupting the normally tight regulation of the HPA axis. Since unbound cortisol passes through the blood-brain-barrier with high efficiency (Pariante et al., 2004) and is known to exert effects on the hippocampus (Sapolsky, 2000), this is another way cortisol and cytokines may interact to affect hippocampal morphology/physiology.

We have outlined some potential mechanisms that could underlie the statistical interactions that we observe. However, it is important to note that our data alone is not sufficient to support any of these mechanisms. Therefore, follow up studies are needed to test the validity of these hypothesized mechanisms. Furthermore, additional brain regions outside the hippocampus could have similar relationships with cortisol and pro-inflammatory cytokines. These relationships will need to be investigated to determine if these findings are specific to the hippocampus.

The current study is not without its limitations. First, we had a small sample size which likely reduced power to detect all significant relationships. Second, our study is cross-sectional, thus the directionality of the relationships we observe is currently unknown and potentially bidirectional. Thus, with these data alone we cannot determine cause and effect relationships between hippocampal volume changes, cytokines, and cortisol. Longitudinal investigations are required to best address these issue of directionality and order of causation. Third, using MRI it is not possible to distinguish between processes that result in cell death and processes that result in changes in morphology. Necrotic and apoptotic processes may underlie the hippocampal volume changes that we observe to be associated with the interactions of cortisol and cytokines. However, other cellular processes such as dendritic atrophy or other cellular morphology changes may also be responsible (Gould et al., 1990; Woolley et al., 1990; Hook et al., 2007). Fourth, while participants in this study were healthy older adults, we did not assess body mass index or other physiological measures concurrently with our measures of HPA and immune function. This could partially limit our ability to detect potential significant findings. Finally, given the absence of any studies examining the interactive effects of cytokines and cortisol levels in this population, we did not have sufficient information to identify a smaller number of cytokine measures for specifying more definitive a priori hypotheses. As such, this is an exploratory, hypothesis generating investigation, which included measures of multiple cytokines. Significance levels obtained here would not withstand Bonferroni correction. Accordingly our results should be interpreted within that context and be used to inform future hypothesis driven studies that cover a narrower scope of observation and address the limitations described here.

In summary, we provide preliminary evidence for interactions between pro-inflammatory cytokine levels, waking cortisol secretion levels, and hippocampal volumes. Specifically we found evidence that higher levels of IL-6 or TNF-alpha in conjunction with higher cortisol levels are associated with smaller hippocampal volumes. Our data suggest that the interaction between waking cortisol and pro-inflammatory cytokines may be as critical an indicator of hippocampal volume-loss as the action of any of these measures in isolation. We suggest that these interactions may be due to either a dual toxicity scenario whereby cortisol and pro-inflammatory cytokines act synergistically on the hippocampus or that cytokines, acting outside of the central nervous system, are driving up levels of unbound cortisol which penetrate the blood-brain-barrier and, over time, cause hippocampal volume changes.

### Conflict of interest statement

The authors declare that the research was conducted in the absence of any commercial or financial relationships that could be construed as a potential conflict of interest.
